# Using Polymers as Crystal Inhibitors to Prevent the Crystallization of the Rotigotine Patch

**DOI:** 10.3390/pharmaceutics16050630

**Published:** 2024-05-08

**Authors:** Qiantong Liu, Xing Li, Bo Liu, Jiahao Kong, Qing Wang, Zhigang Gao

**Affiliations:** 1 School of Chemical Engineering, Dalian University of Technology, Dalian 116024, China; qiant499@163.com (Q.L.); lixing712@163.com (X.L.); 18742515477@mail.dlut.edu.cn (B.L.); kongjh0416@163.com (J.K.); qwang@dlut.edu.cn (Q.W.); 2 Ningbo Institute of Dalian University of Technology, Ningbo 315016, China

**Keywords:** crystal inhibition, polymers, Rotigotine, patch, stability

## Abstract

This study aimed to enhance the stability of the Rotigotine (ROT) patch using polymers as crystal inhibitors. Three polymers (Poloxamer 188, Soluplus, TPGS) were selected as crystal inhibitors to formulate ROT patches with varying drug loadings (20%, 40%, 60%, and 80%, *w*/*w*). SEM and XRD analysis revealed that the Soluplus and Soluplus-TPGS groups with a high concentration (80%, *w*/*w*) of ROT could be stored at room temperature for at least 90 days without crystallization. Moreover, the crystallization nucleation time and growth rate were utilized to assess the ability of Poloxamer 188, Soluplus, and TPGS to hinder the formation of ROT crystals and slow down its crystallization rate. Molecular docking results elucidated the intermolecular forces between ROT and different polymers, revealing their mechanisms for crystal inhibition. The ROT-Soluplus-TPGS combination exhibited the lowest binding free energy (−5.3 kcal/mol), indicating the highest binding stability, thereby effectively reducing crystal precipitation. In vitro skin permeation studies demonstrated that ROT patches containing crystal inhibitors exhibited promising transdermal effects. With increasing ROT concentration, the cumulative drug permeation substantially increased, while the lag time was notably reduced. This study offers novel insights for the development of ROT patches.

## 1. Introduction

Rotigotine (ROT), a non-ergot dopamine agonist (DA), stimulates all dopaminergic (D1-5) receptors, selected serotonergic (5-HT1A) receptors, and adrenergic (α2) receptors. It is commonly used as a first-line treatment for Parkinson’s disease (PD) and restless leg syndrome (RLS) [1,2,3]. Due to its low bioavailability in oral administration, a silicone-based patch (Neupro^®^) was developed for transdermal delivery. Neupro^®^ has been shown to maintain stable and effective plasma concentrations over 24 h, reducing abnormal involuntary movements and demonstrating good efficacy and tolerability in the treatment of PD and RLS [4,5]. However, the unexpected occurrence of polymorphism led to the withdrawal of the patches in the US and Europe in 2008 [6]. Polymorphism, a phenomenon observed in various pharmaceuticals with more than one crystalline form, results from differences in the free energy of the crystalline forms and the solvated state, leading to variations in solubilities and bioavailabilities [7,8,9]. The withdrawal of Neupro^®^ was attributed to the eight-fold lower solubility of the more stable crystalline form II compared to crystalline solid form I, potentially affecting therapeutic outcomes [10].

Crystallization has long been recognized as a critical factor in formulation design, particularly in inhibiting crystallization to ensure therapeutic efficacy and safety [11,12]. For instance, polymers have been used in solid dispersions to prevent recrystallization and enhance physical stability [13,14]. In the case of transdermal patches, maintaining supersaturation of drugs to enhance permeation may increase the risk of crystallization upon storage under incompatible conditions, leading to decreased drug permeation and reduced efficacy of transdermal therapy [15,16,17]. Therefore, strategies to inhibit crystallization play an important role in the development of transdermal patches. Researchers have explored various approaches, such as synthesizing prodrugs to improve solubility, designing transdermal systems with acrylate copolymeric pressure-sensitive adhesives, and utilizing polymers like polyvinylpyrrolidone (PVP) to prevent crystallization of drugs [18,19,20]. 

Medical polymers, which do not possess pharmacological activity but can improve the properties of pharmaceutical preparations, have demonstrated superior biocompatibility and have been used as crystallization inhibitors [21,22,23]. For example, poloxamer 407 (P407) has been shown to stabilize supersaturation with tacrolimus by altering the crystal surface properties [24]. Amphiphilic block copolymers (ABCs) have also gained attention for their surface activity, allowing them to be absorbed onto host crystals [25,26,27,28].

Transdermal administration has been approved to be effective in reducing first-pass metabolism and gastrointestinal side effects, providing continuous release of drugs with stable plasma concentrations, and improving patient compliance [29,30]. Several studies have been conducted on improving stability of ROT patches [31,32,33]. Formulation factors such as the type of pressure-sensitive adhesive, drug loading, and patch thickness have been examined to determine how to enhance the stability of ROT and improve its bioavailability [34,35]. However, investigations on the crystallization inhibition of ROT have been rarely reported, and those particularly based on polymers commonly used as pharmaceutical excipients have not been reported yet. Compared with non-pharmaceutical materials, the use of pharmaceutical polymers as crystallization inhibitors in the preparation of ROT patches not only improves their stability but also effectively reduces their toxic side effects on normal tissues, which is valuable for the clinical application of ROT.

**Scheme 1:**
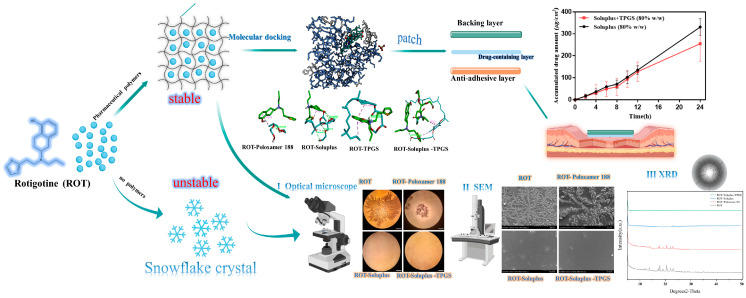
Schematic mechanism illustration for polymer materials inhibiting ROT crystallization.

In this study, we designed ROT patches with polymers commonly used in the pharmaceutical field as crystal inhibitors (Scheme 1). Three polymers (Poloxamer 188, Soluplus, and TPGS) were initially screened through optical microscopy as crystal inhibitors. XRD and SEM were used to investigate the crystal characteristics. The crystal formation time and growth rate were measured by optical microscopy to evaluate the inhibitory ability of the crystal inhibitors. Molecular docking was conducted to analyze the interaction between ROT and polymers to further understand the mechanism of crystallization inhibition. The results of transdermal drug permeation and crystal suppression experiments showed that the Soluplus group and the Soluplus-TPGS group (80%, *w*/*w*) exhibited good stability and transdermal drug permeation performance. This study provides a new strategy to address the crystallization issue in ROT patches.

## 2. Materials and Methods

### 2.1. Materials

Rotigotine (ROT) (Beijing Foyou Pharmaceutical Co., Ltd., Beijing, China); Poloxamer 188 (CAS 9002-96-4, BASF, Ludwigshafen, Germany); Soluplus (CAS 402932-23-4, BASF, Ludwigshafen, Germany); F127 (CAS 9003-11-6, BASF, Ludwigshafen, Germany); HPMC (CAS 9004-65-3, Aladdin, Shanghai, China); PVP (CAS 9003-39-8, Aladdin Shanghai Biochemical Technology, Shanghai, China); TPGS (CAS 9003-39-8, Kunshan Rongbai Biotechnology Co., Ltd., Kunshan, China); Ethyl acetate (CAS 141-78-6, Li’an Longbohua Pharmaceutical and Chemical Co., Ltd., Tianjin, China); Acrylic adhesives, the Durotak^®^ series (Durotak^®^ 87-4098) (Henkel Chemical Co., Zhuhai, China).

Healthy SD rats (SPF, male, 200 ± 20 g) were provided by Liaoning Changsheng Biotechnology Co., Ltd. (Dalian, China).

### 2.2. Methods

#### 2.2.1. Screening of Crystal Growth Inhibitors

ROT (40%, *w*/*w*) was dissolved in a polyacrylic pressure-sensitive adhesive (Durotak^®^ 87-4098). The polymer (HPMC, PVP, F127, Poloxamer 188, Soluplus, and TPGS) were dissolved in 100 μL ethyl acetate, respectively, then added dropwise to the drug-containing pressure-sensitive adhesive and stirred at room temperature for 1 h. The mixtures were poured individually onto glass slides, then dried in a desiccator at 50 °C for 1 h to evaporate organic solvents from the pressure-sensitive adhesive. Afterward, the samples were left at room temperature for 7 days, and the crystallization of ROT was observed using an optical microscope.

#### 2.2.2. Effect of Polymer Ratio on ROT Crystallization

ROT (80%, *w*/*w*) was dissolved in a polyacrylic pressure-sensitive adhesive (Durotak^®^ 87-4098). The screened polymers (Poloxamer 188, Soluplus, and Soluplus-TPGS) were dissolved in ethyl acetate at mass ratios of 1:1, 2:1, 4:1, and 8:1 (ROT:polymer). The subsequent procedures were consistent with the above, and the crystal nucleation time of each group was recorded.

#### 2.2.3. Characterization of ROT Patch

##### X-ray Diffraction (XRD)

To analyze the differences in the ability of different polymers to suppress crystallization, a SMART-LAB X-ray diffractometer (Rigaku, Tokyo, Japan) was utilized to analyze various matrix mixtures. The experiments were conducted with the following parameters: step width of 0.02°; 2θ scanning range from 5° to 50°; generator voltage of 40 kV; generator current of 40 mA; scanning speed of 1°/min.

##### Scanning Electron Microscope (SEM)

To observe the crystalline morphology of ROT, SEM (Zeiss, Oberkochen, German) was employed to examine the surface of different matrix mixtures. Matrix mixtures of ROT with different polymers were cast on glass slides. After the crystals were observed by optical microscope, the samples were coated with gold sputtering and examined at an accelerating voltage of 15 kV.

##### Energy Dispersive X-ray Spectroscopy (EDS)

Energy dispersive X-ray spectroscopy and EDS mapping were used to probe the elemental distribution during ROT crystallization. EDS measurements and mapping were performed at an accelerating voltage of 15 kV. The prepared pressure-sensitive adhesive mixture with ROT was cast on a clean glass plate, then incubated until crystals formed, as described previously. After the crystals formed, the plates were coated with platinum for measurements. The center and edges of each crystal were observed by SEM.

#### 2.2.4. Crystal Inhibition Mechanism

##### Molecular Docking

The molecular structures of ROT, Poloxamer 188, Soluplus, and TPGS were drawn using Chemdraw software 20.0, and Chem3D 14.0.0.17 was used for energy optimization. AutoDock4.2 software [4] was applied to conduct molecular docking experiments. ROT molecules were set as acceptor molecules. The center coordinates of the docking grid were X = −3, Y = −1, Z = 0; the box size was 15 × 15 × 15 Angstroms. Lamarckian genetic algorithms were utilized for molecular docking, and the conformations with the lowest binding free energies were selected for analysis.

##### Determination of Crystal Nucleation Time and Growth Rate

A mixture of ROT and pressure-sensitive adhesive (40%, 60%, and 80%, *w*/*w*) was prepared. Different polymers were added based on ROT: crystal inhibitor (1:1) and cast on glass slides. The crystal nucleation time and growth rate of ROT were monitored using an optical microscope (BX51, Olympus, Tokyo, Japan). The nucleation time of ROT was recorded, and the crystal diameter was measured to calculate the crystal growth rate according to Equation (1):

(1) $$V=\frac{d_{n}-d_{n-1}}{t}$$

where *V*: crystal growth rate (μm/h); *d_n_*: crystal diameter at each time point (μm); *t*: the specified time interval (h).

#### 2.2.5. In Vitro Skin Permeation Test

A mixture of ROT and pressure-sensitive adhesive (20% and 80%, *w*/*w*) was prepared, and crystal inhibitors (Soluplus and TPGS) were added. The Franz diffusion cell method was employed to investigate skin penetration. The anti-adhesive layer of ROT patch was removed, then applied to the stratum corneum of rat skin, and the air bubbles were driven out. The skin was transferred to Franz transdermal diffusion cell (transdermal area of 1.77 cm^2^), with the receiving cell containing 4.0 mL PBS 6.5 (200 rpm, 32 ± 0.5 °C). At predetermined time intervals (2, 4, 6, 8, 10, 12, and 24 h), 1.0 mL of sample was collected, and the receiving medium was replaced with the same volume of fresh PBS. Drug concentration was determined using HPLC after filtration through a polycarbonate membrane (0.45 μm). The cumulative permeated drug amount per unit area (Q) was calculated according to Equation (2):

(2) $$Q=\frac{C_{n}\times V+\sum C_{n-1}\times V_{n}}{A}$$

where *Q*: cumulative permeated drug amount per unit area (μg/cm^2^); *V*: total volume of receiving solution (mL); *C_n_*: measured the drug mass concentration at each time point (μg/mL); *V_n_*: sampling volume (mL); *A*: the diffusion area (cm^2^).

## 3. Results and Discussion

### 3.1. Screening of Potential Inhibitors for Crystal Growth

**Figure 1:**
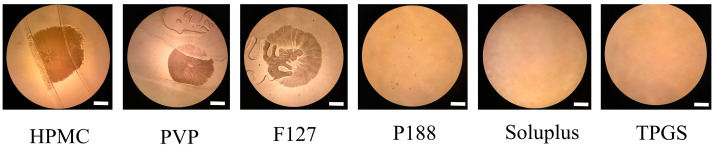
Crystallization of ROT in polyacrylic pressure-sensitive adhesives with different polymers, scale bar = 500 μm.

Optical microscopy was utilized to observe the crystallization of ROT (40%, *w*/*w*) in polyacrylic pressure-sensitive adhesives with various polymers. The crystallization state after 7 days (25 °C ± 2 °C, RH 60% ± 5%) is shown in Figure 1. Snowflake-like crystals of ROT were observed in the HPMC, PVP, and F127 groups. Conversely, crystals were absent in the Poloxamer 188, Soluplus, and TPGS groups, indicating the superior crystal inhibitory ability of these three polymers. Consequently, these three polymers were chosen for subsequent crystallization experiments.

### 3.2. Crystal Morphology Observation of ROT

**Figure 2:**
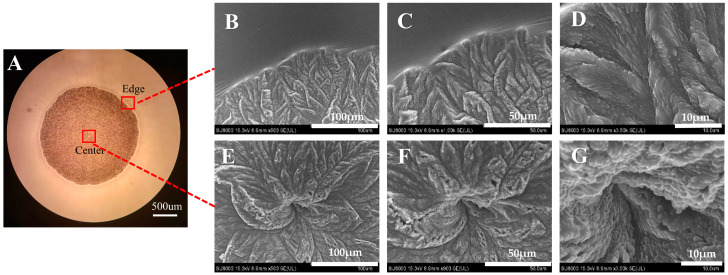
Morphology confirmation of ROT crystals formed in the patch (40%, *w*/*w*); overall photo of ROT crystallization (**A**); SEM-image focusing center (**B**–**D**) and edge (**E**–**G**). Scale bar = 100 μm (**B**,**E**), 50 μm (**C**,**F**), 10 μm (**D**,**G**).

The crystalline morphology of ROT in the pressure-sensitive adhesive was observed using optical microscopy and SEM. As illustrated in Figure 2A, ROT exhibited a spherical crystal shape with a solid nucleus formed by drug aggregation in the center (Figure 2B–D), while dendritic crystals dispersed outward and flocculated crystals were observed at the edges (Figure 2E–G). The crystallization was primarily due to polycrystallization resulting from subtle differences in the arrangement of three-dimensional structure of the longer flexible chains of ROT molecule, leading to a transition from highly soluble crystalline type I to less soluble crystalline type II. Once formed, these crystals continued to grow, enabling more free molecules to form stable crystals, which could impede the transdermal absorption of drug.

### 3.3. Effect of Polymer Ratios on ROT Crystallization

The impact of polymer ratios on ROT crystallization was investigated. As shown in Table 1, in the matrix mixture of ROT (80%, *w*/*w*), as the polymer ratio decreased, the crystallization nucleation time of ROT was shortened to a certain extent. Particularly, the Poloxamer 188 group exhibited the most significant reduction in crystallization nucleation time. Specifically, when the ratio of ROT:Poloxamer 188 reached 8:1, its crystal inhibitory effect was nearly abolished. However, no crystallization was observed in the Soluplus group and the Soluplus-TPGS group at ratios of 1:1 and 2:1 within 30 days. It is worth noting that even when the polymer ratio reached 8:1, the Soluplus group and the Soluplus-TPGS group still exhibited longer crystallization nucleation time (4 days and 8 days, respectively). Additionally, compared to the Soluplus group alone, the crystallization nucleation time of ROT in the Soluplus-TPGS group was significantly prolonged. These findings indicated that the crystal suppression ability of Poloxamer 188 was considerably weaker than that of Soluplus, and the addition of TPGS effectively enhanced the crystal suppression capability of Soluplus.

### 3.4. Characterization of ROT Crystallization in Different Polymers

**Figure 3:**
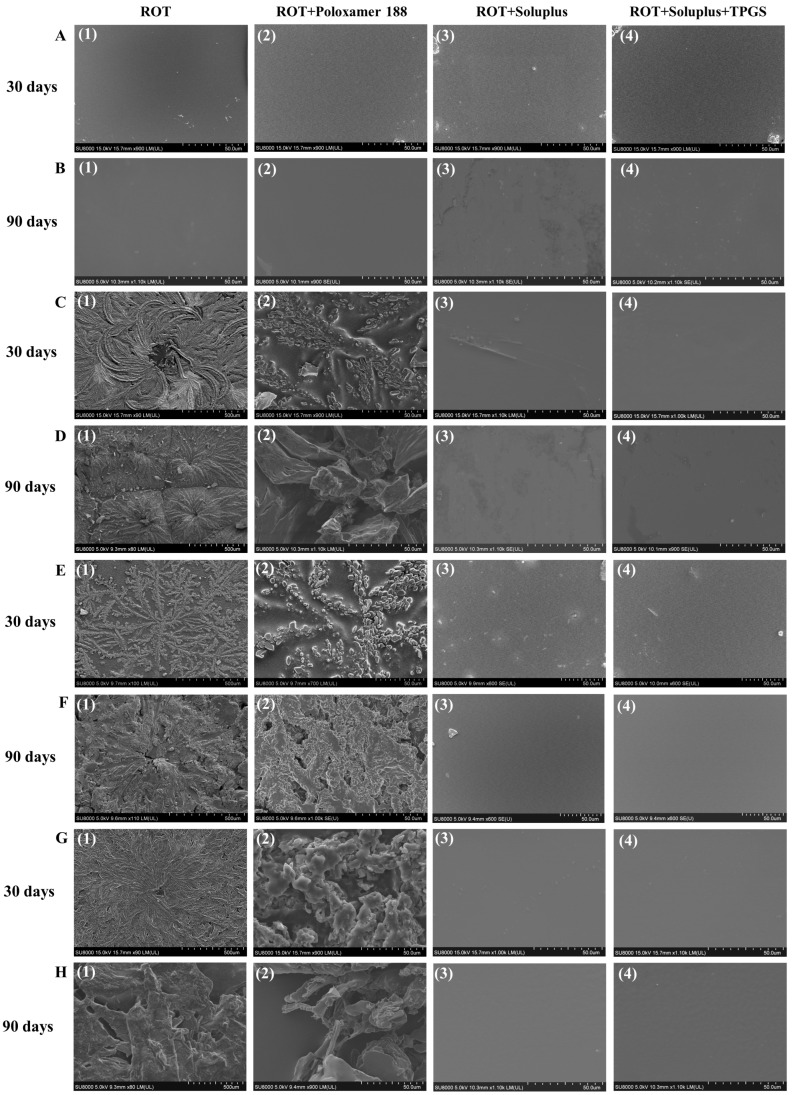
SEM images of ROT crystallization in different polymers. The ROT contents (*w*/*w*) were 20% (**A1**–**B4**), 40% (**C1**–**D4**), 60% (**E1**–**F4**), and 80% (**G1**–**H4**), respectively.

The crystal morphology of ROT in various polymers was examined using SEM and XRD. In the ROT groups (20%, *w*/*w*), no crystals were observed during 90 days (Figure 3(A1–B4)), likely due to ROT not exceeding its saturation solubility in Durotak^®^ 87-4098 pressure-sensitive adhesive, preventing excess ROT molecules from precipitating as crystals. Differences in crystallization appeared between the 40% (*w*/*w*) and 60% (*w*/*w*) ROT groups as the ROT content increased (Figure 3(C1–F4)). Over a 30-day crystallization process, significant crystallization was observed in both the ROT and ROT-Poloxamer 188 groups at 40% (*w*/*w*) and 60% (*w*/*w*), with the ROT-Poloxamer 188 group displaying irregularly dendritic crystallization compared to the snowflake-like crystals in the ROT group, which suggested that Poloxamer 188 can influence the crystalline morphology of ROT. After 90 days, the ROT group at 40% (*w*/*w*) exhibited polycrystalline nuclei growth, while the 60% (*w*/*w*) group showed a large accumulation of crystals. As the ROT content further increased, the ROT group (80%, *w*/*w*) (Figure 3(G1,H1)) and the ROT-Poloxamer 188 group (80%, *w*/*w*) (Figure 3(G2,H2)) showed more obvious ROT crystal accumulation. Notably, no solid crystals of ROT were observed in the ROT-Soluplus group and the ROT-Soluplus-TPGS group at different concentrations (20%, 40%, 60%, and 80%, *w*/*w*) for 90 days, indicating that Soluplus and Soluplus-TPGS had more excellent crystallization inhibition capacity compared to Poloxamer 188.

**Figure 4:**
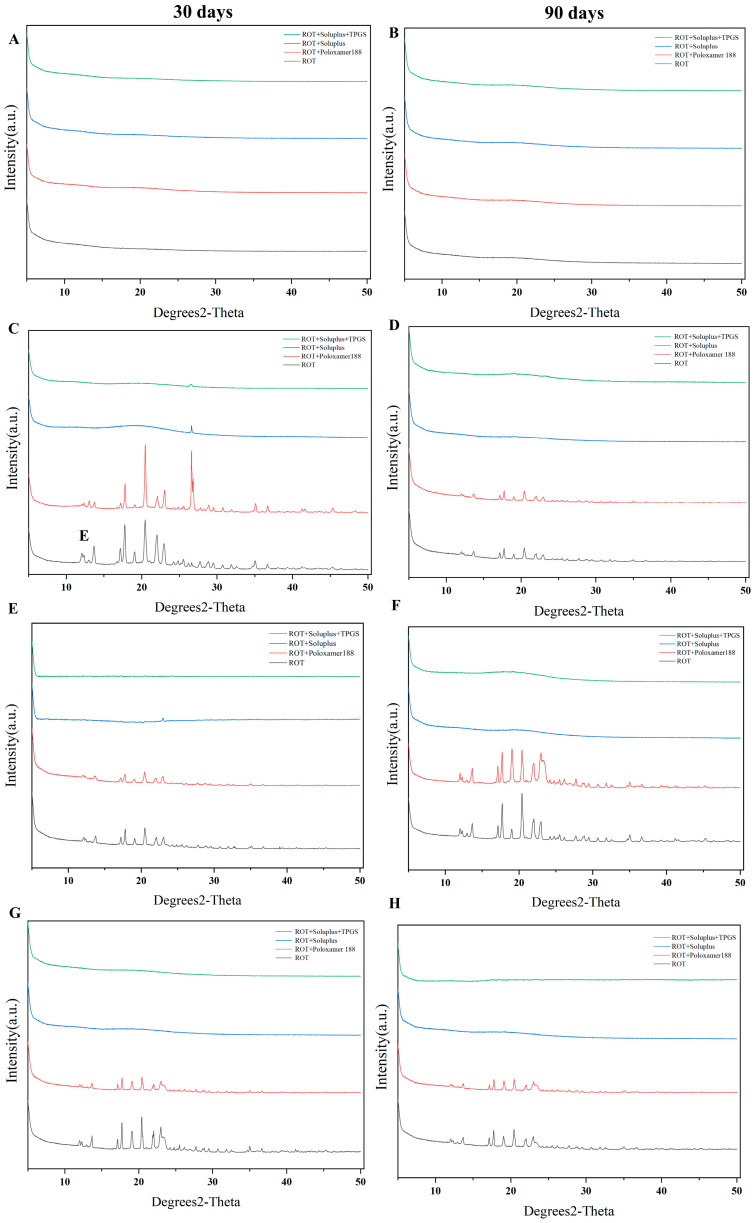
XRD analysis of ROT in different polymers. The ROT contents (*w*/*w*) are 20% (**A**,**B**), 40% (**C**,**D**), 60% (**E**,**F**), and 80% (**G**,**H**), respectively.

The crystallization behavior of different ROT concentration groups was further analyzed using X-ray diffraction (XRD). In Figure 4A,B, no ROT crystal diffraction peaks were observed in the ROT groups (20%, *w*/*w*) after 90 days. In contrast, the ROT groups (40%, 60%, and 80%, *w*/*w*), as well as the ROT-poloxamer 188 group, exhibited distinct ROT diffraction peaks at 2θ angles of 12.089°, 13.034°, 13.664°, 17.130°, 17.812°, 19.072°, 20.595°, 22.013°, 23.010°, 25.531°, 25.736°, 25.531°, 27.736°, 35.140°, and 36.768°, indicating the presence of a significant amount of ROT crystals. Conversely, the ROT-Soluplus and ROT-Soluplus-TPGS groups at various concentrations (20%, 40%, 60%, and 80%, *w*/*w*) did not show any ROT crystal diffraction peaks (Figure 4C–H), suggesting that Soluplus and Soluplus-TPGS effectively inhibited ROT crystallization during the 90-day process. The crystallization inhibitory effects of Soluplus and TPGS to ROT may be related to the fact that Soluplus contains a large number of hydrophilic groups, which can form strong hydrogen bonds with ROT, thereby slowing down the drug recrystallization rate and maintaining the drug in an amorphous state.

**Figure 5:**
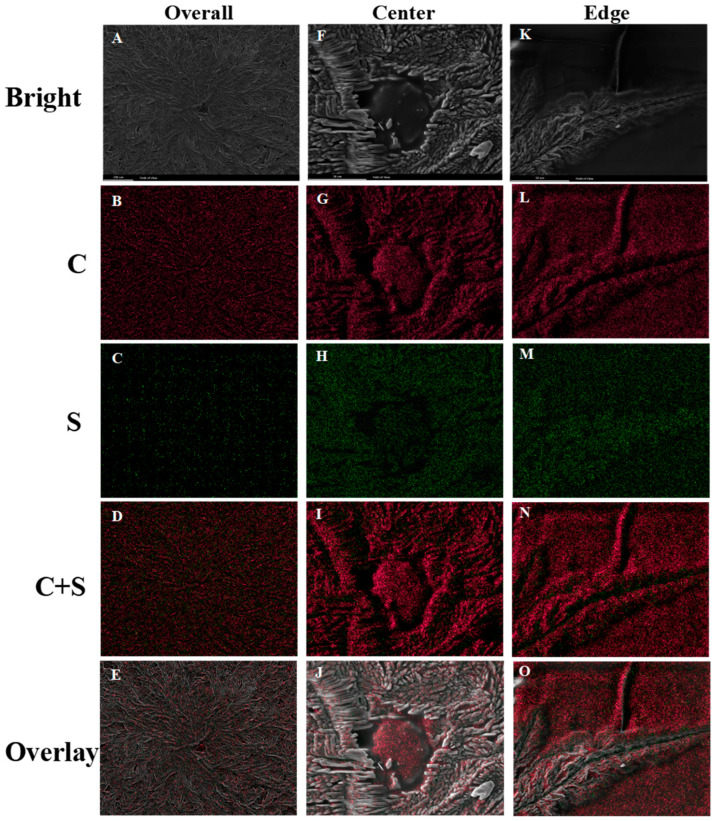
EDS analysis of ROT crystals (80%, *w*/*w*) in polyacrylic pressure-sensitive adhesives ((**A**–**E**): Overall, (**F**–**J**): Center, (**K**–**O**): Edge); C: element Carbon, S: element Sulfur.

**Figure 6:**
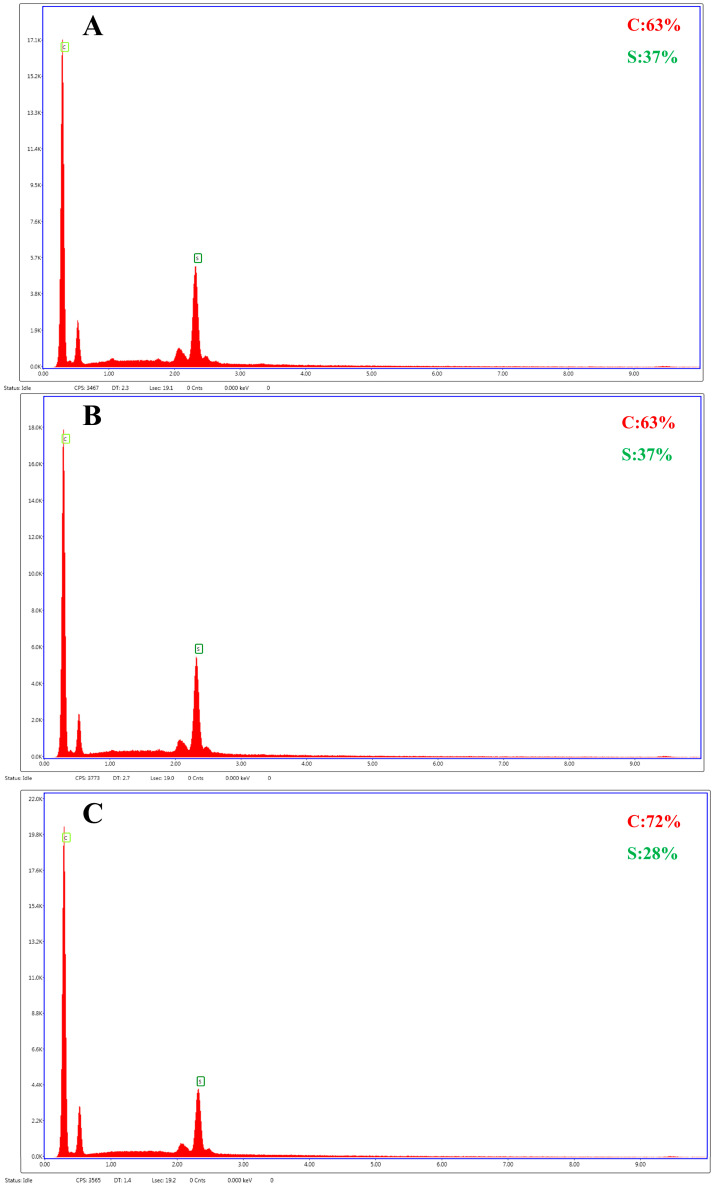
Overall (**A**), center (**B**), and edge (**C**) elemental C and S content of ROT crystals (80%, *w*/*w*) in polyacrylic pressure-sensitive adhesives.

The presence of sulfur in the thiophene group can serve as a specific marker for observing ROT crystals. The EDS analysis of ROT crystallization is presented in Figure 5. The elements carbon and sulfur were evenly distributed throughout the snowflake-like ROT crystals. However, there was a discrepancy in the elemental composition between the central region and the periphery of the ROT crystal. As illustrated in Figure 6, the carbon and sulfur contents in the central region and at the crystallization periphery were 63%, 37% and 72%, 28%, respectively. In comparison to the periphery, the sulfur content in the central region was higher, suggesting that the formation of ROT crystals occurred through the gradual aggregation of oversaturated free ROT molecules to form the central nucleus, which then gradually extended outward to form snowflake-like branches, rather than instantaneously forming irregular crystals with multiple nuclei.

### 3.5. Molecular Docking of ROT with Different Polymers

**Figure 7:**
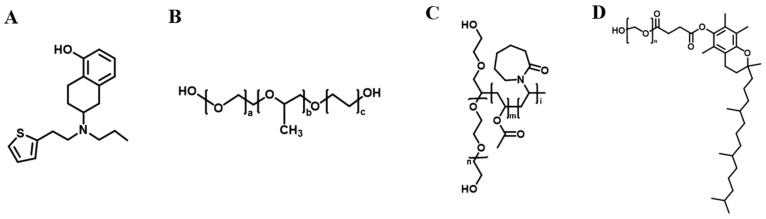
Molecular structures of ROT and different polymers. (**A**) ROT; (**B**) Poloxamer 188; (**C**) Soluplus; (**D**) TPGS.

**Figure 8:**
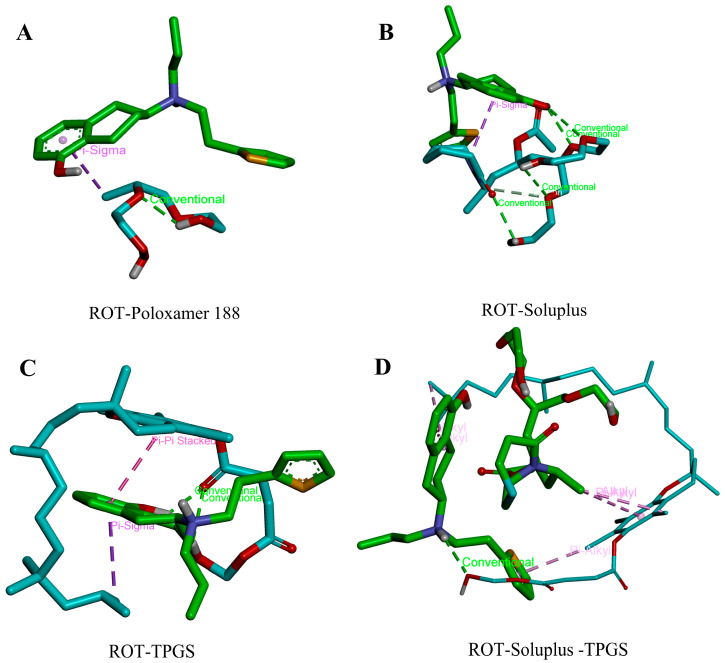
Molecular docking of ROT with different polymers ((**A**): Poloxamer 188, (**B**): Soluplus, (**C**): TPGS, (**D**): Soluplus and TPGS).

To further investigate the mechanism of crystallization inhibition of ROT by three polymers, molecular structures of ROT and different polymers were constructed (Figure 7). Subsequently, molecular docking was performed to explore the interaction between the different polymers and ROT. Theoretical exploration of the interaction force between polymers and ROT was carried out through computer technology (not completely consistent with the actual situation of experimental research). As shown in Figure 8, the dotted lines represent the type of interaction. The green molecules represent ROT, while the cyan molecules represent the polymers. A π-σ stacking interaction was formed between the alkyl C atom of Poloxamer 188 molecule and the benzene ring of ROT. A hydrogen bond was formed between the O atom of Soluplus molecule and the OH of ROT, and a π-σ stacking interaction was formed between the alkyl CH and the benzene ring of ROT. Furthermore, π-π stacking was observed between the aromatic ring of the TPGS molecule and the benzene ring of ROT, with the carbonyl oxygen forming hydrogen bonds with the amino and hydroxyl groups of ROT, and π-σ stacking was formed between the alkyl chain end of TPGS molecule and the benzene ring of ROT. In the ROT-Soluplus-TPGS group, a hydrogen bond was formed between the O atom of the Soluplus molecule and the hydroxyl group of ROT, and a π-σ stacking interaction was observed between the alkyl CH and the benzene ring of ROT. A π-alkyl interaction occurred between the alkyl aromatic ring of TPGS molecule and the methyl group of ROT, with a hydrogen bond formed between the oxygen and amino groups of ROT. Meanwhile, in order to reflect the strength of the intermolecular force, we evaluated the binding ability between ROT and different polymers through the strength of binding free energy. If the binding free energy is positive, external energy may be required during the binding process between the drug and the polymer molecule. If the binding free energy is negative, there is the possibility of docking between drug and polymer molecule, and the lower the binding free energy is, more stable the bond between drug and polymer molecule is [36]. As shown in Table 2, the binding free energies between ROT and polymer were all negative. It showed that the docking between drug and polymer was relatively stable, which could effectively prevent the crystallization of ROT. The ROT-Soluplus-TPGS group exhibited the lowest binding energy (−5.3 kcal/mol), suggesting that TPGS can collaborate with Soluplus to inhibit ROT crystallization effectively.

### 3.6. Evaluation of Crystal Nucleation Time and Growth Rate

Due to the high saturation solubility of ROT in the polyacrylic pressure-sensitive adhesive and high viscosity of the adhesive, the diffusion coefficient of the drug is low. Consequently, immediate drug crystallization is hindered.

**Figure 9:**
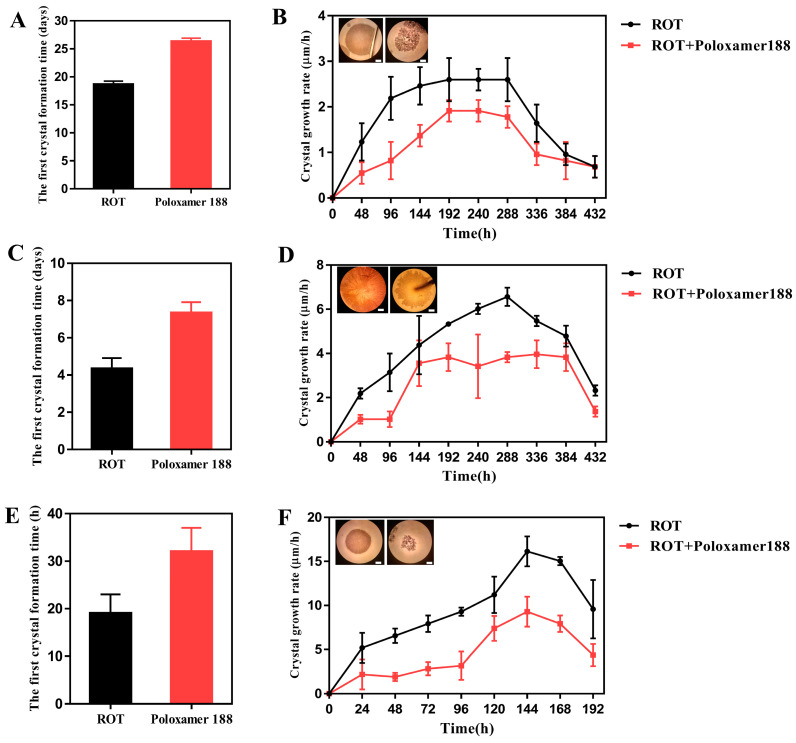
Nucleation time and growth rate for ROT content of 40% (**A**,**B**), 60% (**C**,**D**), and 80% (**E**,**F**) with or without Poloxamer 188 in patches, respectively. Scale bar: 500 µm.

Hence, the establishment of a method for rapid screening of crystal inhibitors is essential [31,37]. Increasing the drug content in the pressure-sensitive adhesive above the saturated solubility threshold promotes rapid drug crystallization. The mixed matrix was cast on a glass slide, which was easy to produce crystals under the influence of external stimuli. No crystallization was observed in the ROT groups with 20% (*w*/*w*) content, indicating no crystal nucleus formation. The crystallization nucleation time of the ROT group (40%, *w*/*w*) and the ROT-Poloxamer 188 group (40%, *w*/*w*) was 19 days and 26 days, respectively (Figure 9A). As shown in Figure 9B, crystal diameters of the ROT group (40%, *w*/*w*) and the ROT-Poloxamer 188 group (40%, *w*/*w*) after 432 h were approximately 3.5 mm and 2.0 mm, and crystals grew very rapidly in the ROT group (40%, *w*/*w*) with a maximum growth rate of 2.60 µm/h. However, the crystal growth rate significantly decreased after addition of the crystal inhibitor Poloxamer 188, reaching a maximum value of 1.91 µm/h.

The crystallization nucleation time of the ROT group (60%, *w*/*w*) and the ROT-Poloxamer 188 group (60%, *w*/*w*) was shortened to 5 days and 8 days, respectively. At the same time, as shown in Figure 9C,D, the crystal diameters of the two groups reached only about 4.0 mm and 2.5 mm at 432 h. The maximum crystal growth rate of the ROT group (80%, *w*/*w*) increased to 6.56 µm/h. After adding the crystal inhibitor Poloxamer 188, the maximum crystal growth rate decreased to 3.97 µm/h. When the content of ROT reached 80% (*w*/*w*), the crystallization of ROT was significantly accelerated. The crystallization nucleation time of the ROT group (80%, *w*/*w*) and the ROT-Poloxamer 188 group (80%, *w*/*w*) were shortened to 22 h and 36 h, respectively (Figure 9E). Meanwhile, the crystal diameters reached 2.0 mm and 1.0 mm after only 144 h. The maximum crystal growth rate of the ROT group (80%, *w*/*w*) increased to 18.04 µm/h. After adding the crystal inhibitor Poloxamer 188, the maximum crystal growth rate decreased to 10.66 µm/h (Figure 9F). Although the crystallization nucleation time and growth rate of ROT were reduced after the addition of crystalline inhibitor Poloxamer 188, the increase in ROT concentration significantly reduced the crystallization inhibition effect of Poloxamer 188. What concerns us most is that the formation of ROT crystal nuclei was not observed in the ROT-Soluplus group and the ROT-Soluplus-TPGS group with a content of 40% (*w*/*w*), 60% (*w*/*w*) and 80% (*w*/*w*) for 90 days. This phenomenon was attributed to the fact that Poloxamer 188, as a traditional medical polymer, can form a π-sigma stacking interaction between the alkyl C atom in its structure and the benzene ring of ROT, but this intermolecular force was weak, limiting its crystallization inhibiting capacity. Therefore, compared with the Soluplus group and the Soluplus-TPGS group, the nucleation time of the Poloxamer 188 group was earlier, and the crystallization rate was also faster. On the contrary, Soluplus, as a large molecular weight medical polymer, can form a large number of hydrogen bonds between the oxygen atoms in its molecule and the hydroxyl groups of ROT. This strong intermolecular force and high viscosity, as well as its long-chain reticular structure, can effectively inhibit the crystallization of the drug, which can greatly prolong the nucleation time of the drug. The crystal inhibitory effect of Soluplus has also been reported in other drugs (indomethacin, glimepiride, fenofibrate, etc.). As a new type of polymer used as pharmaceutical excipients, TPGS is often used in combination with other polymers to synergistically exert a crystallization inhibitory effect [23]. Therefore, the crystallization nucleation time of the Soluplus-TPGS group was the longest. This may be attributed to the presence of multiple intermolecular interactions (hydrogen bonds, π-sigma stacking, and π-alkyl interactions) and the lowest binding free energy (−5.3 kcal/mol) between ROT and Soluplus-TPGS.

### 3.7. In Vitro Skin Permeation Study of ROT Patch

**Figure 10:**
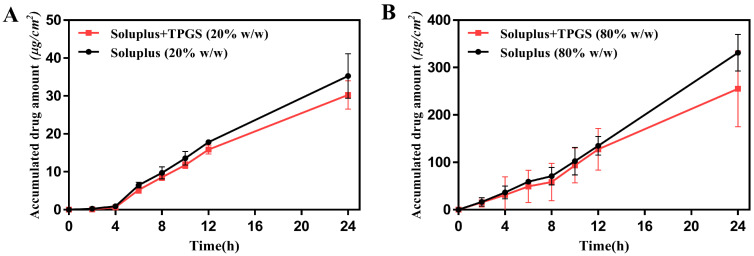
In vitro skin permeation profiles of patches containing 20% (**A**) and 80% (**B**) ROT (*w*/*w*) with different polymers through excised hairless rat skin.

The Franz diffusion cell method was used to measure the skin penetration of ROT. The cumulative penetration amount and permeability of the Soluplus group (20%, *w*/*w*) and the Soluplus-TPGS group (20%, *w*/*w*) in 24 h were 37.62 µg/cm^2^, 31.99 µg/cm^2^ and 1.68 µg/cm^2^/h, 1.44 µg/cm^2^/h, respectively (Figure 10A). When ROT content increased to 80% (*w*/*w*), the cumulative penetration amount and permeability of the Soluplus group (80%, *w*/*w*) and the Soluplus-TPGS group (80%, *w*/*w*) in 24 h increased to 331.13 µg/cm^2^, 255.00 µg/cm^2^ and 13.80 µg/cm^2^/h, 10.63 µg/cm^2^/h, respectively (Figure 10B). As ROT content increased, the penetration effect of patch was significantly improved. Compared with the Soluplus group, the cumulative transdermal penetration percentage of the Soluplus-TPGS group was slightly lower. This may be due to the fact that Soluplus and TPGS interact with one hydrogen bond donor and three hydrogen bond acceptors of ROT, hindering the release of the drug from the pressure-sensitive adhesive. The cumulative permeation amount was in the order of Soluplus > Soluplus-TPGS. It is noteworthy that as ROT content increased, compared with the Soluplus group (20%, *w*/*w*) and the Soluplus-TPGS group (20%, *w*/*w*), for the Soluplus group (80%, *w*/*w*) and the Soluplus-TPGS group (80%, *w*/*w*), the cumulative drug permeation amount in 24 h increased by 7.80 and 6.97 times. This showed that the addition of polymers could effectively inhibit the crystallization of high-concentration ROT and increase the loading capacity of drug to further improve the transdermal permeation efficiency of the patch. At the same time, the lag time for drug permeation was significantly shorter along with ROT concentration increased, which was explained that the diffusion of drugs in the skin was passive and dependent on concentration gradients. According to Fick’s law of diffusion, the higher drug concentration leads to increased dermal permeation as long as it remains below saturation. Furthermore, the presence of free drug on the surface in the highly concentrated patch allows for unhindered transdermal diffusion of free ROT, promoting drug efficacy by quickly penetrating the stratum corneum [38].

## 4. Conclusions

To effectively address the crystallization issue in ROT patches with silicone adhesive, we selected the high-solubility adhesive Durotak^®^ 87-4098 and screened suitable polymers as effective crystal inhibitors. The crystallization of ROT in different polymer groups was characterized by XRD and SEM. In the case of higher drug content (80%, *w*/*w*), no ROT crystals were observed in the ROT-Soluplus (80%, *w*/*w*) group and the ROT-Soluplus-TPGS (80%, *w*/*w*) group after 90 days (25 °C ± 2 °C, RH 60% ± 5%). The molecular docking analysis explored the intermolecular interaction mechanism between different polymers and ROT. The intermolecular binding energy of ROT-Soluplus-TPGS was −5.3 kcal/mol, indicating its effective ability to inhibit crystallization. In vitro transdermal experiments demonstrated favorable drug permeation performance for the Soluplus group (80%, *w*/*w*) and the Soluplus-TPGS group (80%, *w*/*w*). This suggests that polymers, as crystal inhibitors, can effectively prevent the precipitation of supersaturated ROT in the form of crystals, thereby enhancing the stability and efficacy of high-ROT concentration patches. This study provides new insights into crystal suppression in ROT patches.

## Figures and Tables

**Table 1:** Effect of polymers ratio on ROT crystallization.

Polymer:ROT (*w*/*w*)	Samples	Nucleation Time
1:1	ROT	22 h
	Poloxamer188	36 h
	Soluplus	—
	Soluplus-TPGS	—
1:2	ROT	22 h
	Poloxamer188	32 h
	Soluplus	—
	Soluplus-TPGS	—
1:4	ROT	23 h
	Poloxamer188	26 h
	Soluplus	11 d
	Soluplus-TPGS	19 d
1:8	ROT	22 h
	Poloxamer188	22 h
	Soluplus	4 d
	Soluplus-TPGS	8 d

**Table 2:** Molecular binding energy between ROT and different polymers.

Receptor	Ligand	Binding Free Energy (kcal/mol)
ROT	Poloxamer 188	−1.5
	Soluplus	−2.1
	TPGS	−4.0
	TPGS-Soluplus	−5.3

## Data Availability

The original contributions presented in the study are included in the article, further inquiries can be directed to the corresponding author.
